# Production of bio-xylitol from d-xylose by an engineered *Pichia pastoris* expressing a recombinant xylose reductase did not require any auxiliary substrate as electron donor

**DOI:** 10.1186/s12934-021-01534-1

**Published:** 2021-02-22

**Authors:** Tai Man Louie, Kailin Louie, Samuel DenHartog, Sridhar Gopishetty, Mani Subramanian, Mark Arnold, Shuvendu Das

**Affiliations:** 1 Center for Biocatalysis & Bioprocessing, University of Iowa, Iowa City, IA 52241 USA; 2 Department of Chemistry, University of Iowa, Iowa City, IA 52241 USA

**Keywords:** Xylose reductase, Glucose dehydrogenase, Whole cells, *Pichia*, Xylose, And xylitol

## Abstract

**Background:**

Xylitol is a five-carbon sugar alcohol that has numerous beneficial health properties. It has almost the same sweetness as sucrose but has lower energy value compared to the sucrose. Metabolism of xylitol is insulin independent and thus it is an ideal sweetener for diabetics. It is widely used in food products, oral and personal care, and animal nutrition as well. Here we present a two-stage strategy to produce bio-xylitol from d-xylose using a recombinant *Pichia pastoris* expressing a heterologous xylose reductase gene. The recombinant *P. pastoris* cells were first generated by a low-cost, standard procedure. The cells were then used as a catalyst to make the bio-xylitol from d-xylose.

**Results:**

*Pichia pastoris* expressing *XYL1* from *P. stipitis* and *gdh* from *B. subtilis* demonstrated that the biotransformation was very efficient with as high as 80% (w/w) conversion within two hours. The whole cells could be re-used for multiple rounds of catalysis without loss of activity. Also, the cells could directly transform d-xylose in a non-detoxified hemicelluloses hydrolysate to xylitol at 70% (w/w) yield.

**Conclusions:**

We demonstrated here that the recombinant *P. pastoris* expressing xylose reductase could transform d-xylose, either in pure form or in crude hemicelluloses hydrolysate, to bio-xylitol very efficiently. This biocatalytic reaction happened without the external addition of any NAD(P)H, NAD(P)^+^, and auxiliary substrate as an electron donor. Our experimental design & findings reported here are not limited to the conversion of d-xylose to xylitol only but can be used with other many oxidoreductase reactions also, such as ketone reductases/alcohol dehydrogenases and amino acid dehydrogenases, which are widely used for the synthesis of high-value chemicals and pharmaceutical intermediates.

## Background

Xylitol is a five-carbon sugar alcohol that has numerous beneficial health properties [1]. It has almost the same sweetness as sucrose, but with an energy value of 3 cal/g compared to the 4 cal/g of sucrose [2]. Metabolism of xylitol is insulin independent and thus it is an ideal sweetener for diabetics. It also prevents dental caries and ear infection in small children [3]. Therefore, xylitol has found many applications in food products, oral and personal care, and animal nutrition [4, 5]. In addition, U.S. Department of Energy listed xylitol as one of the twelve biobased platform chemicals that can subsequently be used for the synthesis of other high-value chemicals and materials [6]. Xylitol is currently produced by chemical reduction of d-xylose derived from xylan-rich hardwood sources such as birch and beech wood, with a nickel catalyst under high pressure and high temperature with a yield of about 50–60% [7, 8]. The use of metabolically engineered *S. cerevisiae* and *E. coli*, and natural xylitol-producing yeasts such as *Candida* sp. has been considered as an alternative for xylitol production [5]. Biotechnological production processes have the advantages of being safer, more environmentally friendly, and have higher yield and specificity than the chemical reduction. Significant progresses have been made in this area in recent years. For example, Kwon et al. [9] demonstrated a volumetric productivity and xylitol yield of 12 g L^−1^ h^−1^ and 85% (w/w), respectively, from d-xylose with glucose as a co-substrate by cell-recycle fermentation of *Candida tropicalis* in a submerged membrane bioreactor. A metabolically engineered *S. cerevisiae* was constructed to use D-glucose for xylitol production with a 50% yield [10]. Cirino et al. [11] also engineered an *E. coli* that produced 250 mM xylitol from d-xylose with glucose as a co-substrate. In spite of these achievements, biotechnological production of xylitol in industrial scale still faces multiple hurdles. For example, fermentations are usually several days long and other fermentation products are often co-produced with xylitol which complicate downstream processing [12]. In some situations, a portion of the d-xylose is being used to support the growth of the microorganisms instead of xylitol production and thus reduces xylitol yield [13]. Due to industrial scale production and the relative low value of xylitol, using pure d-xylose as substrate in large-scale fermentations is cost prohibitory [12]. Hemicellulose-containing agricultural wastes such as corn cobs and wheat straws are renewable resources and are widely accepted as feasible sources of d-xylose to realize low-cost, biotechnological xylitol production [14]. Dilute acids are commonly used to hydrolyze hemicelluloses and release its sugar constituents [15–18]. However, a broad range of toxic compounds such as furfural, 5-hydroxymethylfurfural, aliphatic acids, and phenolic compounds are co-produced with d-xylose during hydrolysis and inhibit subsequent fermentations. Consequently, detoxification of the hydrolysates prior to being used as fermentation substrates is necessary, which will increase xylitol production cost and time [19].

Here we present a two-stage strategy to produce xylitol from d-xylose using a recombinant *Pichia pastoris* expressing a heterologous xylose reductase gene. Large amount of the recombinant *P. pastoris* cells were first generated by a low-cost, standard procedure. The cells were then used as a biocatalyst to transform d-xylose to xylitol. Our results demonstrated that the biotransformation was very efficient with as high as 80% (w/w) conversion within two hours. The whole cells could be re-used for multiple rounds of catalysis without loss of activity. Also, the cells could directly transform d-xylose in a non-detoxified hemicelluloses hydrolysate to xylitol. Most importantly, we showed that an unknown source of electron donor was present in the *P. pastoris* cells. Oxidation of this unknown source of electron donor could couple with the xylose reductase reaction such that biotransformation of d-xylose to xylitol occurred even when no auxiliary substrate (electron donor) was added to the reaction.

## Results

### Cloning of xylose reductase and glucose dehydrogenase genes into *Pichia pastoris*

**Fig. 1:**
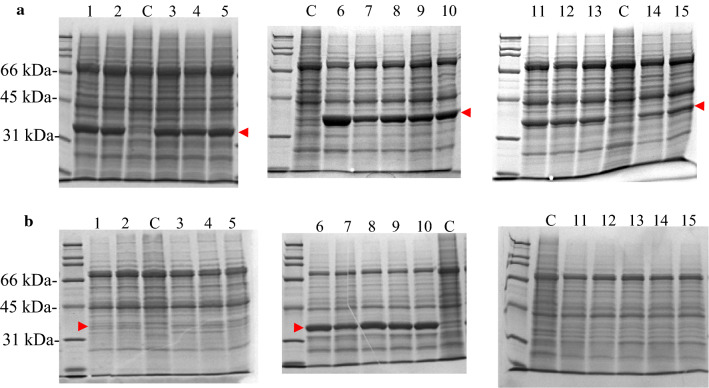
SDS-PAGE gels of cell extracts prepared from various recombinant *P. pastoris* (**a**) expressing XR alone or (**b**) co-expressing XR with GDH. (**a**) Lanes 1 to 5: pPIC4Kx-PsXYL1 clones 1000–1, 1000–2, 4000–1, 4000–2, and 4000–3, respectively. Lanes 6–10: pPIC4Kx-NcXR clones 1000–1, 1000–3, 4000–2, 4000–3, and 4000–6, respectively. Lanes 11–15: pPIC4Kx-CpXr clones 4000–8, 4000–6, 4000–2, 1000–6, and 1000–2, respectively. (**b**) Lanes 1 to 5: pPIC4Kx + PsXLY1 + gdh clones 1000–7, 1000–8, 4000–4, 4000–7, and 4000–8, respectively. Lanes 6 to 10: pPIC4Kx + NcXR + gdh clones 1000–1, 1000–2, 4000–1, 4000–2, and 4000–3, respectively. Lanes 11 to 15: pPIC4Kx + CpXR + gdh clones 1000–5, 1000–6, 4000–4, 4000–5, and 4000–6, respectively. Lane C contained cell extracts of *P. pastoris* GS115 transformed with an empty pPIC4Kx. The red arrows indicated the over-expressed XR proteins

Xylose reductase (XR) genes of *Neurospora crassa* OR47A (*NcXR*), *Candida parapsilosis* ATCC 22019 (*CpXR*), and *Pichia stipitis* CBS 6054 (*PsXYL1)* were cloned and expressed, individually or in combination with the *Bacillus subtilis* glucose dehydrogenase gene (*gdh*) in *Pichia pastoris* GS115. These three XR genes were chosen because of their distinctive preferences for NAD(P)H. NcXR has a strong preference for NADPH, with *k*_*cat*_*/K*_*m, NADPH*_ being 105 times higher than *k*_*cat*_*/K*_*m, NADH*_ [20]. Meanwhile, PsXYL1 can use either NADH or NADPH as co-substrate; its XR activity with NADH was about 70% of that with NADPH [21]. A XR gene (*xyl1*) was previously identified in *C. parapsilosis* KFCC-18075 and the gene product was characterized as an NADH-preferring XR [22]. *C. parapsilosis* KFCC-18075 was reported as a mutant of *C. parapsilosis* ATCC 22019 [23], but the nature of this mutant was not elaborated. Since KFCC-18075 was unavailable, we attempted to amplified *xyl1* (Genbank accession no. AY193716) from genomic DNA prepared from ATCC 22019 using PCR primers designed from *xyl1*. Surprisingly, we failed to amplify any PCR product after numerous attempts. However, using another pair of PCR primers designed from an uncharacterized XR gene (designated as *CpXR* in this report) identified within the genome of *C. parapsilosis* isolate 317 [24], a XR gene was amplified from ATCC 22019 and was successfully expressed in *P. pastoris*. Among the 3 XR genes, *CpXR* expression was the weakest in term of amount of protein and enzyme activity (Fig. 1a, lanes 11–15 and Table 1), while *NcXR* was expressed the best in *P. pastoris*.

**Table 1:** Specific XR, FDH, and GDH activities in cell extracts of selected single recombinant and double recombinant clones (*n* = 3, avg ± SD)

Clone	Specific activity (U/mg protein)		
	XR^a^	GDH^b^	FDH^b^
NcXR 1000–1	62.1 ± 7.1	0.0	0.21 ± 0.03
NcXR 4000–2	35.7 ± 4.0	0.0	0.29 ± 0.03
PsXYL1 1000–1	10.1 ± 0.7	0.0	0.34 ± 0.02
PsXYL14000-3	9.7 ± 1.0	0.0	0.31 ± 0.02
CpXR 1000–6	1.3 ± 0.02	0.0	Not tested
CpXR 4000–8	3.3 ± 0.4	0.0	Not tested
NcXR + GDH 1000–2	22.4 ± 0.8	6.7 ± 0.5	Not tested
NcXR + GDH 4000–1	38.6 ± 1.9	9.2 ± 0.3	Not tested
PsXYL1 + GDH 1000–8	1.7 ± 0. 2	4.4 ± 0.6	Not tested
PsXYL1 + GDH 4000–4	10.2 ± 2.1	17.4 ± 1.0	Not tested
CpXR + GDH 1000–5	0.0	4.6 ± 0.3	Not tested
CpXR + GDH 4000–6	0.0	9.4 ± 0.8	Not tested
pPIC4Kx empty vector control	0.2 ± 0.1	0.02 ± 0.01	Not tested

^a^XR activities were determined with NADPH as electron donor

^b^FDH and GDH activities were determined with NAD^+^ as electron acceptor

*B. subtilis gdh* was co-expressed with the various XR genes in *P. pastoris* such that oxidation of glucose could be used to re-generate NAD(P)H required by the XR reaction. Co-expression of *gdh* with the various XR genes resulted in lower expression levels of all three types of XR. NcXR protein levels in double recombinant clones (Fig. 1b) were lower than the single recombinant clones (Fig. 1a). Distinctive PsXYL1 protein bands were barely visible in the SDS-PAGE gel loaded with cell extracts prepared from *PsXYL1* + *gdh* double recombinant clones, while a distinct CpXR protein band and XR activity were not detectable in cell extracts prepared from *CpXR* + *gdh* double recombinant clones (Fig. 1b and Table 1). Although a distinct 28-kDa band which corresponded to the apparent MW of GDH was not observed in the cell extracts of all double recombinant clones, GDH enzyme activities were detectable in cell extracts (Table 1). Expression levels of XR in our clones, with or without co-expression of GDH, are comparable to other previous reports. For example, *C. guilliermondii xyl1* was expressed in *P. pastoris* at 0.65 U/mg protein [25]. *C. shehatae, C. tropicalis,* and *C. tenuis* XR genes were expressed in *E. coli* at 13.5, 7.8, & 1.8 U/mg, respectively [26–28].

Oxidation of formate by formate dehydrogenase (FDH) is another enzyme reaction commonly used in biocatalysis to regenerate NAD(P)H required by a reductase reaction. Methanol-grown *P. pastoris* produces FDH naturally and the activity was detectable in cell extracts of single recombinant clones with *NcXR* or *PsXYL1* (Table 1). The detected level of FDH activities in our clones are comparable to the value reported in a previously study [29] but are low compared to the XR activities. This imbalance could limit the activity of XR reaction due to insufficient supply of NAD(P)H. Therefore, double recombinant clones with both XR and GDH genes were selected for further study.

### Biotransformation of d-xylose to xylitol by double recombinant cells extracts

Three double recombinant clones, NcXR + GDH 1000–2, NcXR + GDH 4000–1, and PsXYL1 + GDH 4000–4, were chosen for further study because of their high levels of XR and GDH activities (Table 1). When these cell extracts were incubated with 200 mM d-xylose, 100 mM glucose, and 0.25 mM NAD^+^, production of xylitol was detected (Additional file 1: Fig. S1). The PsXYL1 + GDH 4000–4 cell extracts was the most efficient in transforming d-xylose to xylitol, despite having the lowest level of XR activity among the three cell extracts (Table 1). Once d-xylose was mixed with this cell extract, instantaneous formation of xylitol was noticed even in 0 h sample (black trace in Additional file 1: Fig. S1C). Considering the excessive GDH activity in PsXYL1 + GDH 4000–4 cell extracts (GDH to XR activity ratio of 1.7:1), it seems possible that the XR reactions in NcXR + GDH 1000–2 and 4000–1 cell extracts were limited by NAD(P)H supply. However, we did not detect stoichiometric consumptions of glucose in all three reactions (Additional file 1: Fig. S1). *P. pastoris FDH* should be naturally induced in the double recombinant clones when they were grown in BMMY media. We therefore tried coupling of FDH with XR for xylitol production. Xylitol production was detected in reactions with either PsXYL1 + GDH 4000–4 or NcXR + GDH 4000–1 cell extracts (Additional file 2: Fig. S2). Again, PsXYL1 + GDH 4000–4 cell extracts clearly produced xylitol more efficiently. Xylitol was formed immediately after d-xylose was in contact with the cell extracts, resulting in the formation of xylitol in the 0 h samples (black trace in Additional file 2: Fig. S2 A). Surprisingly, xylitol was also produced in reactions with either cell extracts in the absence of formate (Additional file 2 Fig. S2B and D). Xylitol productions in these reactions were definitely catalyzed by enzymatic reactions since boiled cell extracts did not produce any xylitol (data not shown). These results imply the cell extracts contained either an excessive amount of NAD(P)H or an unknown source of electron donor which was continuously oxidized and produced NAD(P)H to sustain the XR reaction.

### Biotransformation of d-xylose to xylitol by double recombinant whole cells

**Fig. 2:**
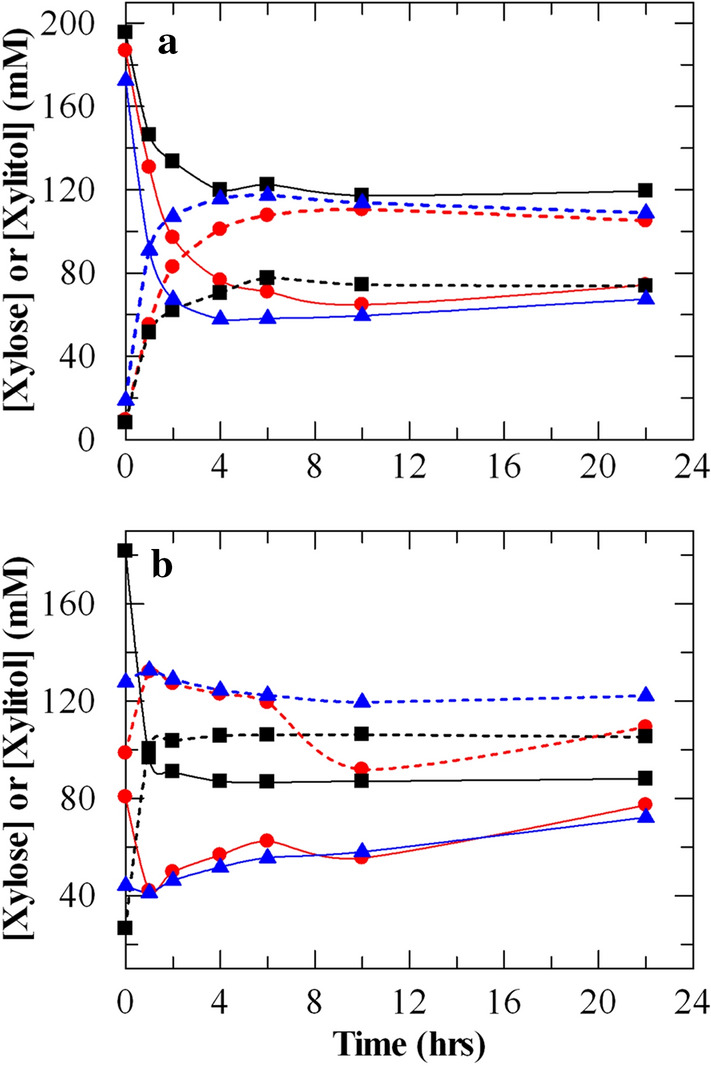
Xylitol production by (**a**) NcXR + GDH 4000–1 and (**b**) PsXYL1 + GDH 4000–4 cells. All reactions had 10 mg/mL of cells. The weight of the cells was a dry cell weight. The cells were incubated at 30 °C with 200 mM d-xylose and 0.25 mM NAD^+^ in 50 mM KPi (pH 7.0) buffer. The reactions also had 100 mM formate (red circle), 100 mM glucose (black square) or no auxiliary substrate as electron donor (blue triangle). Solid lines represent d-xylose consumption; dashed lines represent xylitol production. The data shown in the figure is the average of 3 values. All the values are within the 5% SD

In industrial settings, a whole-cell biocatalytic process is often more preferable than a process that uses cell extracts because the biocatalytic enzymes are protected in a more stable environment. Also, whole cells can be easily recovered and reused for multiple rounds of reactions. Therefore, we tested whole cells of PsXYL1 + GDH 4000–4 and NcXR + GDH 4000–1 clones for xylitol production (Fig. 2). As observed in previous experiments using cell extracts, addition of glucose or formate was not necessary for the XR reaction. In fact, addition of glucose to the reaction mixtures slightly inhibited the XR reaction, resulted in less xylitol. Similar to XR reactions catalyzed by cell extracts, PsXYL1 + GDH 4000–4 whole cells were the most active (Fig. 2); xylitol was detected at the 0 h sample, suggesting immediate transformation of d-xylose to xylitol once the cells contacted with the substrate.

**Fig. 3:**
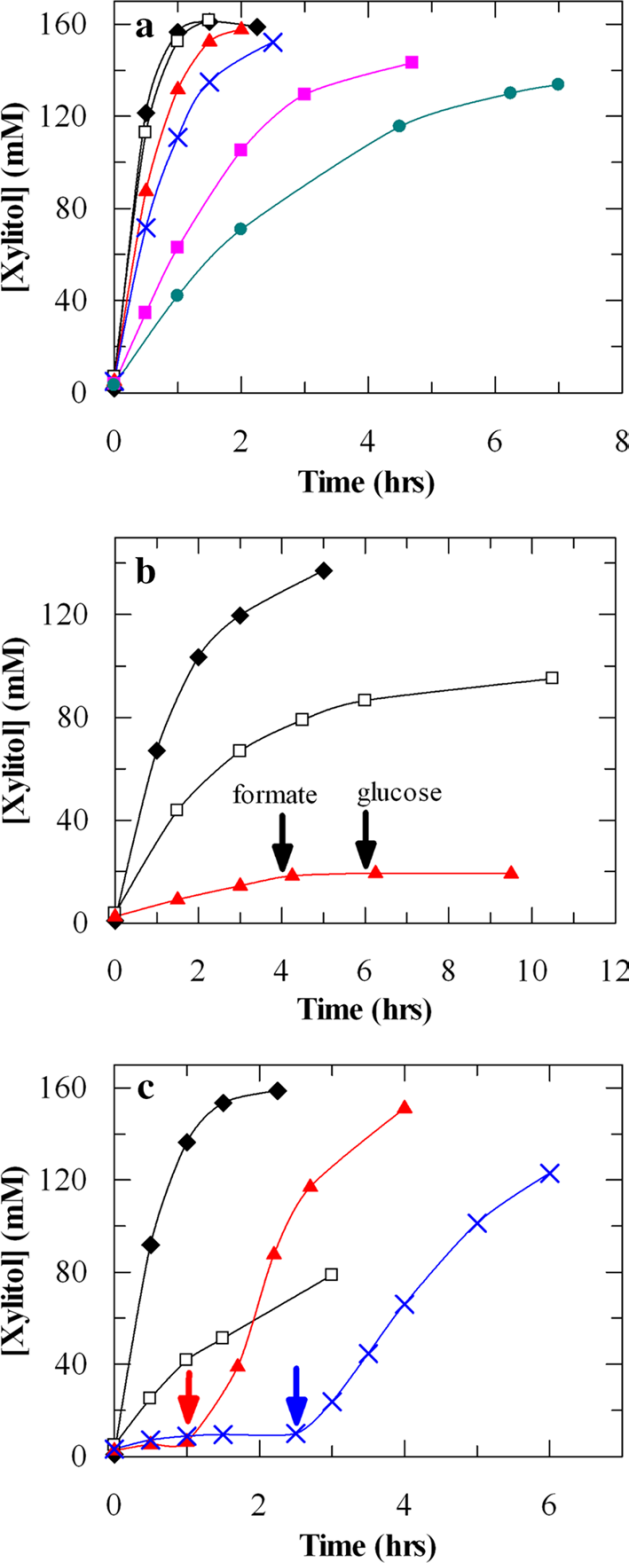
Xylitol production by (**a**, **c**) PsXYL1 + GDH 4000–4 and (**b**) NcXR + GDH 4000–1 cells in multiple cylces of reactions. Symbols: (black diamond) 1st cycle, (open square) 2nd cycle, (red triange) 3rd cycle, (blue cross) 4th cycle, (pink square) 5th cycle, (green circle) 6th cycle. (**a**) 10 mg/mL of PsXYL1 + GDH 4000–4 cells in 50 mM KPi (pH 7.0) buffer were incubated at 30 °C with 200 mM d-xylose and 0.25 mM NAD^+^. At the end of each reaction cycle, the cells were collected by centrifugation and supernatants were removed. Fresh reaction mixture was added to the cells to start another cycle of reaction. (**b**) 10 mg/mL of NcXR + GDH 4000–1 cells in 50 mM KPi (pH 7.0) buffer were incubated at 30 °C with 200 mM d-xylose and 0.25 mM NAD^+^. Recycling was carried out as described above. During the 3rd cycle, 100 mM of formate and glucose were added as indicated. (**c**) 10 mg/mL of PsXYL1 + GDH 4000–4 cells in 50 mM KPi (pH 7.0) buffer were incubated at 30 °C with 200 mM d-xylose but without NAD^+^. NAD^+^ (0.25 mM) was added at the indicated time points (red and blue arrows) in the 3rd and 4th cycles. The data shown in the figure is the average of 3 values. All the values are within the 5% SD

NcXR + GDH 4000–1 and PsXYL1 + GDH 4000–4 cells were recycled in multiple rounds of xylitol production in reaction mixtures containing NAD^+^ but without any auxiliary substrate as electron donor. PsXYL1 + GDH 4000–4 cells could be re-used for at least 6 cycles (Fig. 3a). About 160 mM of xylitol was produced from 200 mM d-xylose within an hour in the first 3 cycles of reaction. On the other hand, xylitol production by NcXR + GDH 4000–1 cells declined significantly after the first cycle of reaction; addition of either glucose or formate could not restore the activity (Fig. 3b). When PsXYL1 + GDH 4000–4 cells were used in a reaction without both NAD^+^ and auxiliary substrate, xylitol production decreased significantly in the second cycle and completely stopped in the third cycle (Fig. 3c). However, the addition of a catalytic amount of NAD^+^ to the reaction mixture immediately restored xylitol production to a rate comparable to fresh PsXYL1 + GDH 4000–4 cells. When these cells were re-used for the fourth time, xylitol was not produced unless NAD^+^ was added again. The results suggest NAD(P)^+^ leached from the PsXYL1 + GDH 4000–4 cells after two cycles of reactions and impaired xylitol production if not being replenished. More importantly, our data indicates rather than having an excessive quantity of NAD(P)H, sufficient amount of an unknown electron donor was present in the cells. Oxidation of this unknown electron donor occurred inside the cells, reducing the added NAD^+^ to NADH, which was then consumed by XR for xylitol production.

**Fig. 4:**
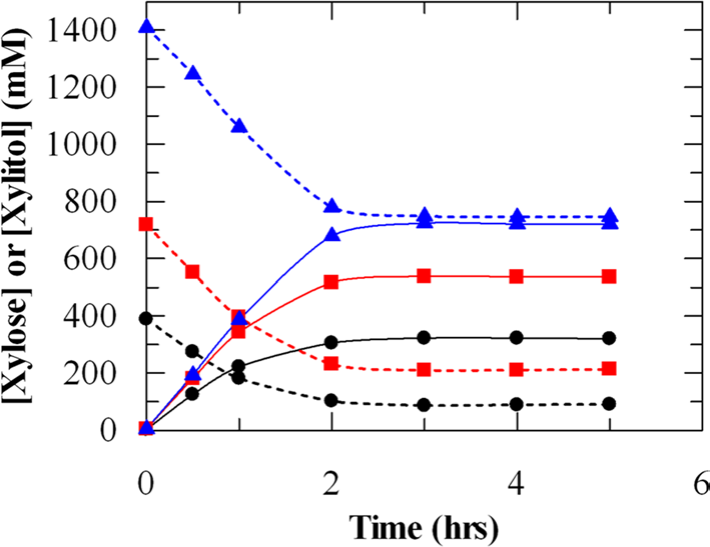
Xylitol production by PsXYL1 + GDH 4000–4 from different d-xylose concentrations. 10 mg/mL of PsXYL1 + GDH 4000–4 cells in 50 mM KPi (pH 7.0) buffer were incubated at 30 °C with 400 mM (black circle), 750 mM (red square), and 1500 mM (blue triangle) of d-xylose. NAD^+^ and auxiliary substrate were not added to the reactions. Solid lines represent xylitol production; dashed lines represent d-xylose consumption. The data shown in the figure is the average of 3 values. All the values are within the 5% SD

We observed about 80% (w/w) conversion of d-xylose to xylitol by PsXYL1 + GDH 4000–4 cells (Fig. 3a, c). The lack of conversion beyond 80% could be due to inhibition by xylitol (i.e. product inhibition) or low concentration of d-xylose near the end of reaction. Multiple NAD^+^- and auxiliary substrate-free reactions were set up by incubating PsXYL1 + GDH 4000–4 cells with various concentrations of d-xylose (Fig. 4). The initial consumption rates of d-xylose were very similar among these reactions, suggesting PsXYL1 in the cells was not inhibited by d-xylose up to 1.5 M. About 320 mM xylitol was produced from 400 mM d-xylose (80% conversion), 535 mM xylitol from 750 mM d-xylose (71% conversion), but only about 750 mM xylitol from 1.5 M d-xylose (50% conversion). The reaction with 1.5 M of d-xylose should not be limited by NAD^+^ since the cells were not recycled. The reaction also should not be limited by electron donor since the same amount of cells supported six cycles of transformation of 200 mM xylose with at least 60% conversion in each reaction cycle (Fig. 3a). The data suggest higher concentration of xylitol could possibly inhibit the XR reaction. It might not be the sole reason why the reaction stopped when 1.5 M d-xylose was used but factors contributed to this inhibitory effect were not clear.

### Biotransformation of d-xylose in a hemicelluloses hydrolysate by PsXYL1 + GDH 4000–4 whole cells

**Fig. 5:**
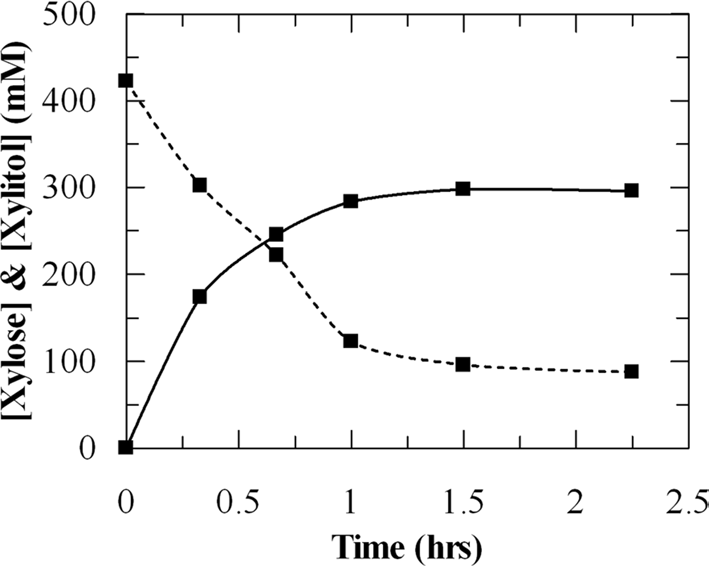
Xylitol production by PsXYL1 + GDH 4000–4 from a hemicelluloses hydrolysate. The PsXYL1 + GDH 4000–4 cells used in this experiment was originated from a 10-L fed-batch fermentation. One L of hemicelluloses hydrolysate (pH 7.0) was incubated with 100 mg/mL PsXYL1 + GDH 4000–4 cells at 30 °C. Solid lines represent xylitol production; dashed lines represent d-xylose consumption. The data shown in the figure is the average of 3 values. All the values are within the 5% SD

We tested the capability of the PsXYL1 + GDH 4000–4 cells to directly transform d-xylose in a non-detoxified hemicelluloses hydrolysate. The hydrolysate contained about 63 g/L (420 mM) d-xylose, 2.0 g/L furfural, and 1.5 g/L hydroxymethylfurfural. Other sugar was not detected in the hydrolysate. The pH of this hydrolysate was slightly acidic and therefore it was adjusted to pH 7.0 before cells (100 mg/mL) were added to 1 L of the hydrolysate solution. The reaction completed within 1 h without the requirement of any NAD^+^ and auxiliary substrate (Fig. 5). About 300 mM xylitol was produced, which was equivalent to 70% (w/w) conversion.

## Discussion

Using cells of a recombinant *Pichia pastoris* expressing the *Pichia stipitis XYL1* and *Bacillus subtilis gdh*, we demonstrated a very efficient biocatalytic process that produced xylitol from d-xylose. The conversion of xylose to xylitol by the xylose reductase is the first step of the pentose-phosphate-pathway dependent xylose metabolism in *Pichia* [30]. This route is very well known for a long time and published in several articles [31, 32]. By using *P. stipitis* xylose reductase, the highest conversion rate obtained was 320 mM xylitol from 400 mM d-xylose (80% conversion) in two hours. The productivity (g of xylitol / g of cells / h of reaction) observed in this transformation was 2.44. This process was also applicable to a non-detoxified hemicelluloses hydrolysate solution containing 420 mM d-xylose, with 300 mM xylitol produced in an hour, with the productivity value of 0.46. Almost one fifth productivity with hemicelluloses hydrolysate compared to the pure xylose, can be attributed to the use of 10 times higher cell concentration and the termination of reaction in 1 h, for the hydrolysate reaction. Ideally, we can’t compare the productivity of pure xylose versus crude hemicellulose hydrolysate because the later contains numerous toxic impurities, such as, furfural, hydroxymethylfurfural, aliphatic acids, and phenolic compounds [33, 34]. The application of whole cells in biocatalysis is not uncommon [35–39]. Whole cells have the capacity to regenerate NAD(P)^+^ cofactor required by oxidoreductase reactions. Moreover, the biocatalytic enzymes are protected in a more stable environment. Production cost reductions are realized in comparison to the use of purified enzymes by recycling the whole cells for multiple rounds of reactions, thereby avoiding the added costs associated with protein purification.

In contrast to xylitol production by fermentations using either natural or metabolically engineered microorganisms, our strategy has several advantages. First, we used a two-stage process that separated fermentation from biocatalysis. Recombinant *P. pastoris* expressing *PsXLY1* and *gdh* were cultivated by a standardized procedure to induce expression of XR and GDH. A large amount of biomass, the biocatalyst, was generated in a short period of time via a 10 L fed-batch fermentation. If we scale up our fermentation process by 100 fold, the per unit production costs of the biocatalyst will be significantly reduced [40]. The cost analysis for the generation of biocatalyst considering scale-up and recycling has been shown in the supplementary information. The procedures to optimize fermentation and recombinant protein expression in *P. pastoris* are well described [41]. Second, reduction of d-xylose to xylitol is extremely efficient. The reaction completed within two hours without the need of NAD^+^ and any auxiliary substrate as an electron donor (Figs. 2, 5). If a catalytic amount of NAD^+^ was included in the reaction mixture, the PsXYL1 + GDH 4000–4 cells could be used for multiple cycles of reaction (Fig. 3a). Recycling up to six cycles, produced a total 904 mM of xylitol with a productivity of 1.15, where only 200 mM of xylose was used in each cycle (Fig. 3a). Sixth cycle produced 82.5% of xylitol (132 mM), compared to the production of first cycle, where 160 mM xylitol produced in the first cycle was considered as 100%. So, there is a still possibility that cells can be reused beyond the sixth cycle (Fig. 3a). Higher concentration (1.5 M) of xylose produced 750 mM of xylitol with a productivity of 5.70 (Fig. 4). If we observe similar recycling trend compared to the previous one, with this higher concentration of xylose, six cycles could produce 4238 mM of xylitol with a productivity of 5.37. Our data also showed that these cells could directly transform d-xylose in a non-detoxified hemicelluloses hydrolysate to xylitol, with efficiency similar to reactions that used pure d-xylose as substrate (Fig. 5). Non-detoxified hemicelluloses hydrolysates could not be used as a d-xylose source in xylitol-producing fermentation processes with natural or metabolically engineered microorganisms because furfural, hydroxymethylfurfural, aliphatic acids, and phenolic compounds in the hydrolysates inhibit the growth of the microorganisms [19]. Apparently, these inhibitors do not affect PsXYL1 enzyme activity in the cells. We hypothesize PsXYL1 + GDH 4000–4 cells contained an internal source of elector donor, which was oxidized to generate NADH required by the XR reactions (Figs. 2, 3, and 4). In whole-cell biocatalytic processes involving oxidoreductases, an enzyme system (such as GDH and FDH) that oxidizes an auxiliary substrate (like glucose or formate) is normally used to regenerate NAD(P)H. The PsXYL1 + GDH 4000–4 cells completely bypassed the external addition of the auxiliary substrate. Production cost is reduced further because separation of the co-product generated from the auxiliary substrate (such as gluconolactone) from xylitol is not necessary. As there is no role of GDH for this biocatalytic production of xylitol, use of a single recombinant clone expressing XR altogether could be the subject of a follow up investigation, from an academic standpoint.

It has been previously reported that bio-reduction catalyzed by yeasts could be carried out without any auxiliary substrate as electron donor. In one case, reduction of the carbonyl group of acetoacetate occurred when about 5 mg yeast per mL (mixture of *S. cerevisiae* and *S. bayanus*) was used to catalyze the reaction [42]. In another report, reduction of the C = C double bond of (*E*)-3-ethyl-4-(3-pyridyl)-3-buten-2-one by Baker’s yeast happened without the requirement of any auxiliary substrate as electron donor when a larger amount of yeast was used [43]. Both reports proposed certain unidentified substance stored inside the yeast cells might be used as electron donors for generating NADPH required by the oxidoreductases. It is known that glucose, glucan, and triacylglycerols are often stored in yeast cells [44–46]. Such accumulated molecules produced by the metabolic pathways inside the yeast cells, can serve as an electron donor for the regeneration of NAD(P)H from NAD(P)^+^. To our knowledge, there is no previous study which examined the storage compounds accumulation inside *P. pastoris*. Over-expression of heterologous proteins in *P. pastoris* could pose stressful conditions to the cells and might induce accumulation of storage compounds. The complete genome sequences of two different strains *P. pastoris* were recently available for identification of genes and metabolic pathways [47, 48]. A search of *P. pastoris* GS115 genome database [49] using keywords such as glycogen and acyltransferase easily identified genes that could contribute to triacylglycerol synthesis (locus ID chr2-1_0694 and chr3_0218) and degradation (chr2-1_0794), as well as genes that could be involved in glycogen accumulation (locus ID chr1-3_0035 and chr3_0781) and degradation (locus ID chr2-2_0437). These findings suggest *P. pastoris* possibly possesses the enzymes and metabolic pathways to synthesize storage compounds inside the cells.

The biocatalytic process reported here can be improved in several areas. First, we noticed that there was sufficient NAD(P)^+^ inside the cells to support the XR reaction. However, the cell recycling experiment showed that these internal NAD(P)^+^ leached out from the cells and brought the XR reaction to a complete halt (Fig. 3c). Addition of a catalytic amount of NAD^+^ immediately restored the reaction. Therefore, if NAD(P)^+^ that leached out from the cells could be separated from the product stream and recycled back to the next round of reaction, we would realize multiple cycles of xylitol production by simply incubating the cells with d-xylose. The isolation of NAD with ≥ 98% recovery from a biocatalytic reaction mixture, has been demonstrated by using ion-exchange chromatographic methods [50, 51]. This same strategy should be able to purify NAD(P)^+^ from the product stream.

This biocatalytic process could also be optimized via adjusting the cells to substrate ratio, reaction temperature, and pH control. Moreover, complete genome sequence of *P. pastoris* is available and thus modification of the genetic background of the *P. pastoris* to improve NADH regeneration has become feasible [52]. It will also be possible to improve the accumulation of the currently unknown electron donor via metabolic engineering once the nature of this unknown electron donor is identified.

## Conclusions

We demonstrated here that the cells of a recombinant *P. pastoris* expressing *XYL1* from *P. stipitis* and *gdh* from *B. subtilis* could transform d-xylose, either in pure form or in crude hemicelluloses hydrolysate, to xylitol very efficiently. This biocatalytic reaction happened without the requirement of any NAD(P)^+^, NAD(P)H, and auxiliary substrate as an electron donor. A significant amount of an electron donor, whose identity is currently unclear, appears to be present inside the cells and oxidation of this compound re-generated NAD(P)H required by the xylose reductase. The findings reported here have a broader impact and are not limited to the conversion of d-xylose to xylitol. Many oxidoreductase reactions, such as ketone reductases/alcohol dehydrogenases or amino acid dehydrogenases, are used for the synthesis of high-value chemicals and pharmaceutical intermediates [36, 53–55]. Our experimental design and findings might be applied to and improved these industrially relevant processes.

## Methods

### Materials

All chemical reagents were purchased from Sigma-Aldrich (St. Louis, MO) and Fisher Scientific (Pittsburgh, PA) unless otherwise noted. PCR primers were purchased from Integrated DNA Technologies (Coralville, IA). Turbo *Pfu* DNA polymerase and corresponding buffer (Stratagene, La Jolla, CA) were used in all PCR reactions unless otherwise noted. Other molecular biology reagents were purchased from Invitrogen (Carlsbad, CA), New England Biolabs (Ipswich, MA), Fermentas (Glen Burnie, MD), Promega (Madison, WI), Qiagen (Valencia, CA), and Epicentre (Madison, WI). Hemicellulose hydrolysate was provided by Sriya Innovation, Inc. (Marietta, GA).

### *Pichia pastoris* expression plasmid

*P. pastoris* expression plasmid pPIC3.5K (Invitrogen) was modified for expression of xylose reductase and glucose dehydrogenase gene. First, the *Afe*I site at nucleotide position 1524 of pPIC3.5K was removed by site-directed mutagenesis using primers pPIC35sdm-F and pPIC35sdm-R (Additional file 3: Table S1) with the Quikchange II site-directed mutagenesis kit from Stratagene (La Jolla, CA). The resultant plasmid was designated as pPIC3.5Kx. Plasmid pPIC3.5Kx was sequentially digested by *Afe*I and *Bst*Z17I and yielded two blunt-ended DNA fragments (7911 and 1093 bp). The 7,911-bp DNA fragment was gel-purified and self-ligated to form pPIC4Kx and was used in all subsequent cloning experiment.

### Cloning of the *Bacillus subtilis* glucose dehydrogenase gene (gdh)

Plasmid pUC19-gdh was a generous gift from Dr. Jack Rosazza (Univ. of Iowa). The *gdh* gene sequence contained an internal *Asu*II restriction site which interfered with subsequent cloning procedures. Therefore, site-directed mutagenesis was used to remove this internal *Asu*II site (without changing protein sequence) with primers gdh-sdm-F and gdh-sdm-R (Additional file 3: Table S1). After that, the mutated *gdh* gene was amplified by PCR using primers gdh-F and gdh-R (Additional file 3: Table S1). The PCR product was gel-purified, followed by *Asu*II and *Eco*RI digestion, and ligated into pPIC4Kx pre-digested with *Asu*II and *Eco*RI, forming plasmid pPIC4Kx-gdh. DNA sequencing confirmed successful cloning of the mutated *gdh* into pPIC4Kx, with no mutation introduced in the coding sequence due to cloning procedures.

### Cloning of the *Pichia stipitis* xylose reductase gene (PsXYL1)

Genomic DNA of *Pichia stipitis* CBS 6054 was purchased from American Type Culture Collection (ATCC 58785D-2) and was used as template for PCR amplification of the *PsXYL1* gene with primers PsXYL1-F and PsXYL1-R (Additional file 3: Table S1). The PCR product was gel-purified, followed by *Asu*II and *Mfe*I digestion, and ligated into pPIC4Kx pre-digested with *Asu*II and *Eco*RI (*Eco*RI cut site is compatible with *Mfe*I cut site), forming plasmid pPIC4Kx-PsXYL1. DNA sequencing confirmed successful cloning of the *PsXYL1* gene into pPIC4Kx with no mutation introduced due to the cloning procedures.

### Cloning of the *Candida parapsilosis* xylose reductase gene (CpXR)

*C. parapsilosis* ATCC 22019 was cultivated in YM broth (Becton, Dickinson and Company, Sparks, MD) and genomic DNA was extracted from the culture using Puregene yeast genomic DNA purification kit (Qiagen). An uncharacterized xylose reductase gene *(CpXR)* was identified in the genome of *Candida parapsilosis* isolate 317 [18]. A pair of degenerate PCR primers CpXR-F2 and –R2 (Additional file 3: Table S1) were designed based on this uncharacterized *XR* gene and successfully amplified a 1-kb PCR product from genomic DNA prepared from ATCC 22,019 using FailSafe PCR buffer G (Epicentre) in combination with *Taq* DNA polymerase (New England Biolabs). The PCR product was directly cloned into PCR product cloning vector pGEM-Teasy (Promega). DNA sequencing confirmed the resultant plasmid, pGEM-Teasy + CpxR F2R2B6, contained *CpXR* identified in *C. parapsilosis* isolate 317 genome. PCR primers CpXR-F3 and CpXR-R3 (Additional file 3: Table S1) were used to amplify the *CpXR* from pGEM-Teasy + CpxR F2R2B6 using FailSafe PCR buffer G and *Taq* DNA polymerase. The PCR product was gel-purified, followed by *Asu*II and *Eco*RI digestion. The restriction digested PCR product was ligated into plasmid pPIC4Kx pre-digested with *Asu*II and *Eco*RI, forming plasmid pPIC4Kx-CpXR.

### Cloning of the *Neurospora crassa* xylose reductase gene (NcXR)

*NcXR* is (Genbank accession no. NW_001849801.1) composed of 3 exons which are 142, 791, and 486 bp in size. Coding sequences of *NcXR* are located on exon 1, exon 2, and the first 36 nucleotides of exon 3. So, the complete *NcXR* ORF is 969 nucleotides in length. We used the crossover PCR technique described by Link et al. [56] to create in-frame fusion of exons 1 and 2. A *N. crassa* cosmid clone, G1-F11, that contained the *NcXR* gene was purchased from the Fungal Genetics Stock Center at the University of Missouri, Kansas City. Forward primer Ex1out (Table S1) was designed in region 5′ to the ATG start codon of *NcXR* in exon 1. In combination with reverse prime Ex1in (Additional file 3: Table S1), a PCR product that contained exon 1 DNA sequence was amplified from cosmid G1-F11 using Turbo *Pfu* DNA polymerase. Another pair of primers Ex2in and Ex2out (Table S1) were used to amplify exon 2 from cosmid G1-F11. These 2 PCR products were mixed together with primers Ex1out and Ex2out for amplification of an exon 1-exon 2 in-frame fusion product by the crossover PCR procedure. The PCR product was designated as PCR #F. PCR #F was gel purified and was used as template for a regular PCR using primers NcXR-F and Ex1&2in (Additional file 3: Table S1) with Turbo *Pfu* DNA polymerase. This 0.9-kb PCR product was gel purified and used as template in a final round of PCR with primers NcXR-F and Ex123-R (Additional file 3: Table S1). This final PCR product was ligated into pGEM-Teasy, forming pGEMT-easy NcXR and DNA sequencing confirmed successful cloning of *NcXR*. The *NcXR* was released from pGEM-Teasy NcXR using *Asu*II and *Eco*RI (*Asu*II site engineered on primer NcXR-F. *Eco*RI site located on pGEM-Teasy plasmid, 10 nucleotides 3′ to the stop codon of *NcXR*). This *Asu*II-*Eco*RI fragment was ligated into plasmid pPIC4Kx pre-digested with *Asu*II and *Eco*RI, forming plasmid pPIC4Kx-NcXR.

### Construction of expression plasmid with both XR and GDH expression cassettes

In all the *P. pastoris* expression plasmids, the gene of interest (either *XR* or *gdh*) is flanked by an *AOX1* promoter (*P*_*AOX1*_) and the *AOX1* transcription terminator (*AOX*_*TT*_). The *P*_*AOX1*_*-gdh-AOX*_*TT*_ expression cassette was released from pPIC4Kx-gdh by *Bam*HI and *Bgl*II digestion. This cassette was cloned into pPIC4Kx-PsXYL1 and pPIC4Kx-CpXR at the *Bam*HI site, forming plasmids pPIC4Kx-PsXYL1-gdh and pPIC4Kx-CpXR, respectively. The *gdh* expression cassette was cloned into the *Bgl*II site of pPIC4Kx-NcXR, producing pPIC4Kx-gdh-NcXR.

### Transformation of *Pichia pastoris* GS115 with expression plasmids

Plasmids pPIC4Kx-PsXYL1-gdh, pPIC4Kx-CpXR-gdh, pPIC4Kx-gdh-NcXR, pPIC4Kx-PsXYL1, pPIC4Kx-CpXR, pPIC4Kx-NcXR, and pPIC4Kx were linearized by *Bsp*EI before electroporation. The linearized plasmids were individually transformed into electrocompetent *P. pastoris* GS115 prepared according to the procedure reported by Wu and Letchworth [57]. The transformed cells were then plated on minimal dextrose-sorbitol agar (1.34% yeast nitrogen base without ammonium and amino acids, 4 × 10^–5^% biotin, 2% dextrose, 1 M sorbitol, and 2% agar) plates and incubated at 30 °C for 5–7 days. Expression plasmids integrated into GS115 genome would render a His^+^ phenotype to the transformants. His^+^ transformants that grew on minimal dextrose-sorbitol agar were pooled together and plated on YPD agar (1% yeast extract, 2% peptone, 2% dextrose, and 2% agar) containing 250, 500, 1000, 1500, 2000, 3000, and 4000 µg/ml of geneticin to screen for His^+^ transformants with multiple copies of expression plasmids integrated into the GS115 genome. Colonies that grew on YPD-geneticin (1000 µg/ml) and YPD-geneticin (4000 µg/ml) plates are termed as “NcXR 1000” and “NcXR 4000”, respectively. After the geneticin concentration, the numbers such as, 1 and 2 were used to identify the specific colonies on the respective YPD-geneticin plates. Colonies grown on YPD-geneticin plates were streaked for purity on minimal dextrose-sorbitol agar plates. After obtaining single colonies, they were transferred back to YPD-geneticin agar to ensure the isolated colonies were resistant to high concentration geneticin before chosen for protein expression study.

### Protein expression study of selected transformants

Five transformants generated from each expression plasmid were chosen for protein expression study. Among the five transformants, two were resistant to 1 mg/mL of geneticin and three were resistant to 4 mg/mL geneticin. A single colony of each transformant was used to inoculate 20 mL BMGY broth (1% yeast extract, 2% peptone, 100 mM potassium phosphate (pH 6), 1.34% yeast nitrogen base without ammonium and amino acids, 4 × 10^–5^% biotin, and 1% glycerol). The cultures were incubated at 30 °C for 16 h with orbital shaking at 300 rpm. In the next day, the BMGY cultures were used to inoculate 40 mL BMMY broth (same as BMGY except 0.5% methanol replaced 1% glycerol) in 500-mL baffled flasks. Methanol in the BMMY broth served as carbon/energy source for the cells as well as the inducer for protein expression. The BMMY cultures were incubated at 30 °C for 48 h with orbital shaking at 300 rpm. After 24 h, methanol was added to the BMMY cultures to a final concentration of 0.5% to maintain induction. At 24 and 48 h, 1 mL of cells was sampled from each culture for measuring the cell density and protein expression levels. At 48 h, all the cells in each culture were harvested by centrifugation at 4,000×*g* for 5 min. The 1-mL cell samples collected at 24 and 48 h post induction were re-suspended in 196 µL of Y-PerR Plus yeast protein extraction reagent (Thermo Scientific), 2 µL 0.5 M EDTA, and 2 µL 100X Halt Protease inhibitor cocktail (Thermo Scientific). To each cell pellet, an equal volume of glass beads (0.5 mm) was also added. The glass beads-cells suspensions were then vortexed vigorously for 30 s and then immediately chilled on ice for 30 s. This vortex-chilling procedure was repeated 7 more times. After that, the whole suspensions were incubated at 45 °C for 15 min with shaking at 300 rpm. Finally, the suspensions were centrifuged at 13,000 rpm for 2 min and the supernatants were loaded onto 10% SDS-PAGE gels (Bio-Rad) for analyses of XR and GDH expressions.

### Fed-batch fermentation of *P. pastoris* PsXYL1 + GDH 4000-4

Fed-batch fermentation was conducted by following a modified procedure that was originally described by Zhang et al. [41]. Fermentation was done at 10 L scale to obtain biomass containing xylose reductase and glucose dehydrogenase. Inoculum was grown to an optical density of 1.5 in shake flasks with medium consisting of 15.5 g/L glycerol, 5 g/L ammonium sulfate, 1.5 g/L yeast nitrogen base (YNB), and 0.16 mg/L biotin. Fermentation medium contained 10 g/L glycerol, 3.5 g/L ammonium sulfate, 4.7 g/L corn steep, and P2000 antifoam. Fermentation conditions were: 30 °C, pH of 5 controlled with 4 M sodium hydroxide and 25 LPM airflow with agitation increasing from 300 to 800 rpm to maintain 30% dissolved oxygen. The glycerol concentration was monitored, and glycerol (50.0 g/L) & corn steep (18.3 g/L) feed was initiated when glycerol levels fell below 2 g/L (at 18 h). Glycerol and corn steep were used to increase the cell biomass where they were acted as a carbon source and nitrogen source supplements, respectively. When OD at 595 nm reached over 100 at 32 h, 60 mL methanol was added to the fermentor to start induction of xylose reductase and glucose dehydrogenase. The glycerol & corn steep feed was stopped 30 min after methanol addition. Methanol concentration was monitored, and methanol feed was initiated to maintain concentrations between 2 and 10 g/L. At 7 h post-induction, YNB and biotin feed was started, with a total addition of 9 g YNB and 2.7 mg biotin over 10 h. Samples were withdrawn at different time intervals and OD, concentration of glycerol & methanol were measured. Cells (2.67 kg) were harvested at 63 h of elapsed fermentation time and stored at – 80º C until further use.

### Enzyme activity assays

The major cell pellets harvested from BMMY cultures were suspended in 7 mL 50 mM KPi buffer (pH 6.0) with 70 µL 100X Halt Protease inhibitor cocktail, and 7 µL 1 M dithiothreitol. Each cell suspension was lysed by passing through a chilled French Press cell twice at 138 MPa. Unbroken cells and cell debris were removed from the lysate by centrifugation (22,000×*g* for 20 min at 4 °C). The clear supernatant was designated as cell extracts. XR activity assay was carried out at 30 °C in 50 mM KPi buffer (pH 6) containing an appropriate amount of cell extracts, 200 mM d-xylose, and 0.2 mM of NADPH. The reaction was initiated by the addition of NADPH to the reaction mixture. XR enzyme activity was determined by monitoring the decrease in absorbance at 340 nm (Δε_340_ = 6,220 M^−1^ cm^−1^) due to NADPH consumption. One unit of XR activity was defined as the consumption of 1 µmole of NADPH per min under the defined conditions. Formate dehydrogenase (FDH) activity assay was carried out at 35 °C in 50 mM KPi buffer (pH 7.5) containing an appropriate amount of cell extracts, 100 mM ammonium formate, and 1.5 mM of NAD^+^. The reaction was initiated by the addition of ammonium formate to the reaction mixture. FDH enzyme activity was determined by monitoring the increase in absorbance at 340 nm due to NADH production. One unit of FDH activity was defined as the production of 1 µmole of NADH per min under the defined conditions. Glucose dehydrogenase (GDH) activity assay was carried out at 30 °C in 50 mM KPi buffer (pH 7.5) containing an appropriate amount of cell extracts, 100 mM glucose, and 1 mM of NAD^+^. The reaction was initiated by the addition of glucose to the reaction mixture. GDH enzyme activity was determined by monitoring the increase in absorbance at 340 nm due to NADH production. One unit of GDH activity was defined as the production of 1 µmole of NADH per min under the defined conditions.

### Biotransformation of d-xylose to xylitol by whole cells

All d-xylose-to-xylitol biotransformation reactions that used pure d-xylose as substrate were incubated at 30 °C in 50 mM KPi (pH 7) buffer with 10 mg/mL cells and 200 mM d-xylose. The total reaction volume was 5 mL. NAD^+^ (0.25 mM), glucose (100 mM) and formate (100 mM) were added when specified. In the recycling experiment in which cells were reused in multiple rounds of reaction, reactions were scaled up tenfold (50 mL final volume). After each round of reaction, cells were collected by centrifugation (4,000×*g*, 5 min) and re-suspended in 50 mL fresh reaction solution for the next cycle of biocatalysis. When hemicelluloses hydrolysate was used directly as substrate, the pH of the hemicelluloses hydrolysate was first adjusted to approximately 7.0 with a 5 M sodium hydroxide solution. One liter of the pH adjusted hydrolysate solution was then incubated with 100 mg/mL cells at 30 °C with orbital shaking at 200 rpm. In all experiments with cells, aliquots of reaction mixtures were removed from the reactions at various time points. Solids in the samples were removed by centrifugation at 13,000 rpm for 2 min, followed by filtration through a 0.22-µm filter. The cell-free supernatants were analyzed by for xylitol production and d-xylose consumption by a high performance liquid chromatography (HPLC) system.

### Analytical procedures

Identification and quantification of d-xylose and xylitol were conducted with a Shimadzu LC-10AD HPLC system equipped with a photodiode array detector and a Shimadzu RID-10A refractive index detector. Separation of compounds was achieved on an Aminex HPX-87H column (Bio-Rad, 300 × 7.8 mm). The column was maintained at 30 °C during operation. Sulfuric acid (5 mM) was used as a mobile phase with a flow rate of 0.6 mL/min. HPLC peak identifications were established by comparing the compounds’ retention times with those of authentic standards. Protein concentrations in cell extracts were determined by Bradford assay (Bio-Rad) with bovine serum albumin as standard. Protein bands on SDS-PAGE gels were visualized after staining with GelCode Blue staining reagent (Thermo Scientific). The weight of the cells was expressed as a dry weight in this study. After harvesting the cells, the pre-weighed wet cells were placed in an oven and dried at 90 °C until a stable weight was reached. One hundred gram of wet cells produced 26.2 g of dried cells under the above conditions used. Xylitol productivity was calculated based on the amount of xylitol produced in gram by each gram of dry cells in every hour of reaction, for a specific biotransformation. For the recycling experiments, as we reused the cells, we added all the yields and reaction times from each cycle whereas amount of cells used was considered only from the 1st cycle. The percentage of bioconversion of xylose to xylitol was calculated based on the molar concentrations of xylose used at the beginning of the reaction and xylitol produced at the end of the reaction in a specific bioconversion.

## Supplementary Information

**Additional file 1: Figure S1.** Biotransformation of D-xylose to xylitol by cell extracts of (**A**) NcXR+GDH 1000-2, (**B**) NcXR+GDH 4000-1, and (**C**) PsXYL1+GDH 4000-4. Enzyme reactions in (A) and (B) had 13 and 24 U of NcXR activity, respectively. Reaction in (C) had 5.4 U of PsXYL1 activity. Cell extracts was incubated at 30oC with 200 mM D-xylose, 100 mM glucose, and 0.25 mM NAD+ in 50 mM KPi (pH 7.0) buffer. Glycerol (10%, v/v) was added to all reactions for stabilizing enzyme activities. At 0 hr (black), 2 hr (blue), and 12 hr (red), samples were drawn from each reaction for HPLC analyses.

**Additional file 2: Figure S2.** Biotransformation of D-xylose to xylitol by cell extracts of (A & B) PsXYL1+GDH 4000-4 and (C & D) NcXR+GDH 4000-1 with or without formate. Enzyme reactions in (A & B) had 2.7 U of PsXYL1 activity while reactions in (C & D) had 12 U of NcXR activity. Cell extracts was incubated at 30oC with 200 mM D-xylose and 0.25 mM NAD+ in 50 mM KPi (pH 7.0) buffer. Formate (100 mM) was present only in the reactions in (A & C).

**Additional file 3: Table S1.** PCR primers used in this study.
