# Supplementary material for: Production of bio-xylitol from d-xylose by an engineered Pichia pastoris expressing a recombinant xylose reductase did not require any auxiliary substrate as electron donor
Source: Microb Cell Fact. 2021 Feb 22;20:50. doi: 10.1186/s12934-021-01534-1 (PMC7898734; doi:10.1186/s12934-021-01534-1)
Supplement: Supplementary file 1 — Additional file 1: Figure S1. Biotransformation of D-xylose to xylitol by cell extracts of (A) NcXR+GDH 1000-2, (B) NcXR+GDH 4000-1, and (C) PsXYL1+GDH 4000-4. Enzyme reactions in (A) and (B) had 13 and 24 U of NcXR activity, respectively. Reaction in (C) had 5.4 U of PsXYL1 activity. Cell extracts was incubated at 30oC with 200 mM D-xylose, 100 mM glucose, and 0.25 mM NAD+ in 50 mM KPi (pH 7.0) buffer. Glycerol (10%, v/v) was added to all reactions for stabilizing enzyme activities. At 0 hr (black), 2 hr (blue), and 12 hr (red), samples were drawn from each reaction for HPLC analyses. [file 12934_2021_1534_MOESM1_ESM.pptx]

## Slide 1
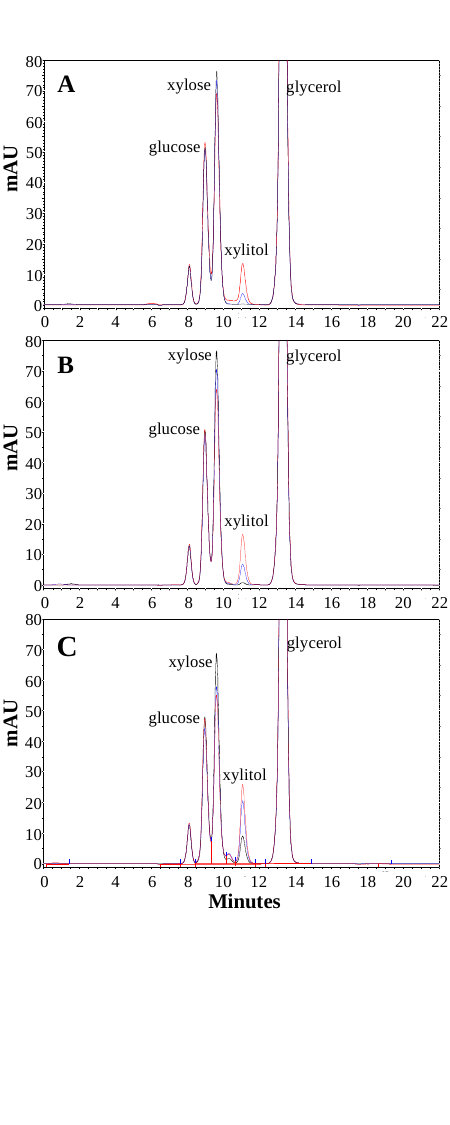

70
0
3
10
20
22
8
9
1
2
0
7
8
8
12
8
14
16
8
18
8
5
6
4
8
80
70
60
50
40
30
20
10
0
3
10
20
22
8
9
1
2
0
7
8
8
12
8
14
16
8
18
8
5
6
4
8
80
70
60
50
40
30
20
10
0
3
10
20
22
8
9
1
2
0
7
8
8
12
8
14
16
8
18
8
5
6
4
8
A
xylose
glycerol
glucose
mAU
xylitol
B
xylose
glycerol
glucose
mAU
xylitol
C
glycerol
xylose
glucose
mAU
xylitol
Minutes
80
60
50
40
30
20
10
