# Supplementary figures and images for: Production of bio-xylitol from d-xylose by an engineered Pichia pastoris expressing a recombinant xylose reductase did not require any auxiliary substrate as electron donor
Source: Microb Cell Fact. 2021 Feb 22;20:50. doi: 10.1186/s12934-021-01534-1 (PMC7898734; doi:10.1186/s12934-021-01534-1)

## Slide 1
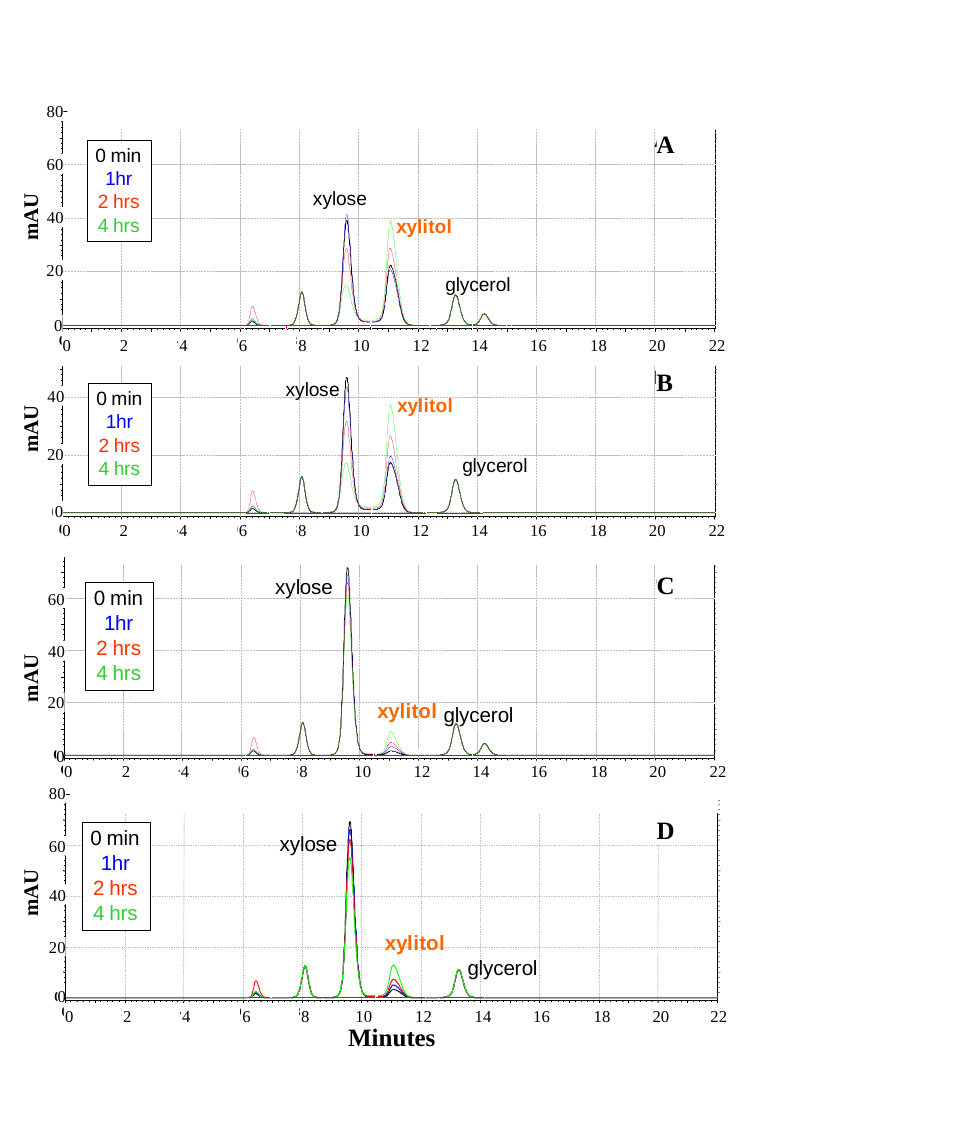

80
60
40
20
0
A
mAU
3
10
20
22
8
9
1
2
0
7
8
8
12
8
14
16
8
18
8
5
6
4
8
B
40
20
0
mAU
3
10
20
22
8
9
1
2
0
7
8
8
12
8
14
16
8
18
8
5
6
4
8
80
60
40
20
0
C
mAU
3
10
20
22
8
9
1
2
0
7
8
8
12
8
14
16
8
18
8
5
6
4
8
80
60
40
20
0
D
mAU
3
10
20
22
8
9
1
2
0
7
8
8
12
8
14
16
8
18
8
5
6
4
8
Minutes

Supplement: Supplementary file 2 — Additional file 2: Figure S2. Biotransformation of D-xylose to xylitol by cell extracts of (A & B) PsXYL1+GDH 4000-4 and (C & D) NcXR+GDH 4000-1 with or without formate. Enzyme reactions in (A & B) had 2.7 U of PsXYL1 activity while reactions in (C & D) had 12 U of NcXR activity. Cell extracts was incubated at 30oC with 200 mM D-xylose and 0.25 mM NAD+ in 50 mM KPi (pH 7.0) buffer. Formate (100 mM) was present only in the reactions in (A & C). [file 12934_2021_1534_MOESM2_ESM.pptx]
