# Supplementary material for: Production of bio-xylitol from d-xylose by an engineered Pichia pastoris expressing a recombinant xylose reductase did not require any auxiliary substrate as electron donor
Source: Microb Cell Fact. 2021 Feb 22;20:50. doi: 10.1186/s12934-021-01534-1 (PMC7898734; doi:10.1186/s12934-021-01534-1)
Supplement: Supplementary file 3 — Additional file 3: Table S1. PCR primers used in this study. [file 12934_2021_1534_MOESM3_ESM.docx]

**Table S1: PCR primers used in this study**

|  |  |
| --- | --- |
| Primer | DNA sequence* |
|  |  |
|  |  |
| pPIC35sdm-F | 5'-GTCACTATGGCGTGCTGCTGGATCCATATGCGTTGATGCAATTTC-3' |
| pPIC35sdm-R | 5'-gaaattgcatcaacgcatatggatccagcagcacgccatagtgac-3' |
| gdh-sdm-F | 5'-GCCTGGCTTGCTTCCAAGGAAGCCAGCTA-3' |
| gdh-sdm-R | 5'-tagctggcttccttggaagcaagccaggc-3' |
| gdh-F | 5'-CGCGCGTTCGAACAAAATGTACCCGGATTTAAAAGG-3' |
| gdh-R | 5'-GAATTAGAATTCTTAACCGCGGCCTGCCTGGA-3' |
| PsXR-F | 5'-GCGCGCTTCGAACAAAATGCCTTCTATTAAGTTGAA-3' |
| PsXR-R | 5'-GGCGAGCAATTGTTAGACGAAGATAGGAATCT-3' |
| CpXR-F2 | 5'-ATGTCNATYAARTTRAAYTCNGG-3' |
| CpXR-R2 | 5'-CTARACAAARAYTGGAATGT-3' |
| CpXR-F3 | 5'-CGCGGCTTCGAACAAAATGTCGATTAAATTAAATTC-3' |
| CpXR-R3 | 5'-TAAGCTGAATTCCTAGACAAAGATTGGAATGTGATC-3' |
| Ex1out | 5'-ACGCAGTGAGGGGACAACATGAGCCGAAGT-3' |
| Ex1in | 5'-CTCAACCTCGTTGCCGTAGTCGCAGGCACCATCGAAGAGG-3' |
| Ex2in | 5'-CCTCTTCGATGGTGCCTGCGACTACGGCAACGAGGTTGAG-3' |
| Ex2out | 5'-GTTGGTGGGCTGGTTGAAGCGGATGCC-3' |
| NcXR-F | 5'-GCGCGCTTCGAACAAAATGGTTCCTGCTATCAAGCT-3' |
| Ex1&2in | 5'-AGGTTCTCAGCGGAGAAGTAGTTGGTGGGCTGGTTGAAGCG-3' |
| Ex123-R | 5'-ctaaccgaaaatccagaggttctcagcggagaagta-3' |
|  |  |

*Underlined sequences represent restriction enzyme cut sites engineered into the primers.

**Cost Analysis for Generation of Biocatalyst:**


Cost of 10-L in-house fed-batch fermentation was $4800 including the raw materials and harvest. We have shown that these cells can be recycled at least 6 times without loss of significant activity, which effectively reduces the cost of biocatalyst to $800 per cycle. The sixth cycle produced 82.5% of xylitol compared to the production of the first cycle, which leaves additional activity beyond the sixth cycle, thereby further reducing the production cost on a per cycle basis. Additional costs per cycle can be realized by producing the cells at a 1000 L pilot scale fermentation at a cost of $10,800. Assuming a corresponding 100-fold increase in cell production, the cost per cycle drops to $18 compared to $800 for the cells produced at a 10 L scale. Beyond 6 cycles, the effective unit cost of the cells is reduced further.
